# Non-idiopathic peripheral facial palsy: prognostic factors for outcome

**DOI:** 10.1007/s00405-020-06398-6

**Published:** 2020-10-06

**Authors:** Katharina Geißler, Elisabeth Urban, Gerd F. Volk, Carsten M. Klingner, Otto W. Witte, Orlando Guntinas-Lichius

**Affiliations:** 1 Department of Otorhinolaryngology, Jena University Hospital, Am Klinikum 1, 07747 Jena, Germany; 2 Facial Nerve Center, Jena University Hospital, Jena, Germany; 3 Department of Neurology, Jena University Hospital, Jena, Germany

**Keywords:** Facial nerve, Paralysis, Paresis, Prognosis, Electrodiagnostics, Stapedius reflex, Recovery

## Abstract

**Objectives:**

There is a lack of data on patients’ and diagnostic factors for prognostication of complete recovery in patients with non-idiopathic peripheral facial palsy (FP).

**Methods:**

Cohort register-based study of 264 patients with non-idiopathic peripheral FP and uniform diagnostics and standardized treatment in a university hospital from 2007 to 2017 (47% female, median age: 57 years). Clinical data, facial grading, electrodiagnostics, motor function tests, non-motor function tests, and onset of prednisolone therapy were assessed for their impact on the probability of complete recovery using univariable and multivariable statistics.

**Results:**

The most frequent reason for a non-idiopathic peripheral FP was a reactivation of Varicella Zoster Virus (VZV; 36.4%). Traumatic origin had a higher proportion of complete FP (52.9%). Furthermore, in traumatic FP, the mean interval between onset and start of prednisolone therapy was longer than in other cases (5.6 ± 6.2 days). Patients with reactivation of VZV, Lyme disease or otogenic FP had a significant higher recovery rate (*p* = 0.002, *p* < 0.0001, *p* = 0.018, respectively), whereas patients with post-surgery FP and other reasons had a significant lower recovery rate (*p* < 0.0001). After multivariate analyses voluntary activity in first EMG, Lyme disease and post-surgery cause were identified as independent diagnostic and prognostic factors on the probability of complete recovery (all *p* < 0.05).

**Conclusion:**

Infectious causes for non-idiopathic FP like VZV reactivation and Lyme disease had best probability for complete recovery. Post-surgery FP had a worse prognosis.

**Level of evidence:**

2

**Electronic supplementary material:**

The online version of this article (10.1007/s00405-020-06398-6) contains supplementary material, which is available to authorized users.

## Introduction

Peripheral facial palsy (FP) is a most common cranial nerve damage presented to otolaryngologists, neurologists, general practitioners, pediatricians. The most frequent diagnosis for FP remains idiopathic FP (Bell’s palsy; 70%) [1], i.e. a diagnosis of exclusion. Bell’s palsy has an incidence of 10–40 per 100.000 [2]. The complete recovery rate for Bell’s palsy after onset of prednisolone treatment within 72 h in the two milestone phase III trials was about 85–94% within 9–12 months [3, 4].

In contrast, less is known on the outcome of non-idiopathic FP. Temporal bone fractures and facial wounds are a common cause for traumatic FP (10–23%, [1]). Furthermore, iatrogenic lesions during otologic, parotid and vestibular schwannoma surgery are reasons for postoperative FP [1].

Ramsay Hunt syndrome (4.5–7%) due to geniculate ganglionitis by reactivation of Varicella Zoster Virus (VZV) is another common diagnosis of non-idiopathic peripheral FP. Outcome is much worse than in Bell palsy [5].

Acute otitis media, cholesteatoma and necrotizing otitis externa can also cause FP [1]. An important cause is Lyme disease. In endemic areas, Lyme disease causes up to 10–25% of all cases of FP [6]. To complete, malignant tumors and rare genetic syndromes are other reasons for non-idiopathic FP [1, 7].

Beginning with 2007, the diagnostic and therapeutic procedures including a standardized therapy regime for all patients with acute FP were conformed in the Departments of Otorhinolaryngology and Neurology of Jena University Hospital, Germany. A prospective data registration of these patients was started. In the present study, we analyzed the recovery rates for the different causes and identified prognostic factors of complete recovery.

## Methods

### Ethical considerations

The institutional ethics committee approved the study protocol for a data collection of routine and anonymized hospital data.

### Study design and patients

A standardized prospective data collection was started in 2007 in the Departments of Otorhinolaryngology and Neurology of Jena University Hospital, Germany. The present study focused on patients with non-idiopathic peripheral FP in the acute phase. All patients received an otorhinolaryngologic examination including ultrasound of head and neck and a neurological examination. Laboratory tests included serum and cerebrospinal fluid (CSF) and were analyzed for IgM and IgG antibodies against borrelia, varicella zoster virus (VZV), herpes simplex virus (HSV) using enzyme-linked immunosorbent assays (ELISA).

In case of a positive borrelia ELISA, a borrelia IgM and IgG line immunoblot assay was performed. Two or more diagnostic criteria were needed to fulfill the diagnosis of Lyme borreliosis: Recent erythema migrans, borrelia antibodies in serum or CSF, CSF pleocytosis > 5 WBC/mm^3^, CSF/serum index > 1.5.

The presence of mucocutaneous vesicles in the external auditory canal, on the tympanic membrane or on the base of the tongue, or IgM or IgA antibodies against VZV or HSV detected in serum qualified for classification as virally associated FP.

Eight hundred and six patients (805) patients with peripheral FP were admitted between January 2007 and December 2017, i.e. within a period of 11 years. One hundred and seventy-three patients (173), presenting for the first time after > 2 months after onset, were excluded. All patients with Bell´s palsy (368) were excluded. A causal origin was already known or could be found in 264 patients. These patients were included in this study.

All patients were hospitalized. Treatment followed the underlying disease and the German guideline for the treatment of FP [11]. As symptomatic treatment, a tapered course of oral corticosteroids over 7 days was regarded as standard treatment [11]. This conservative treatment was given to all patients. In case of VZV reactivation, the patients received additionally acyclovir for 5 days, in case of Lyme disease ceftriaxone for 7 days.

### Diagnostics and main outcome measures

#### FP grading system

On the day of first presentation, the FP was graded by the admitting physician according to the House-Brackmann (HB) six-point facial grading system [8], and also according to the Stennert index [9, 10]. The Stennert index classifies the face at rest (0–4 points; 0 = normal to 4 = complete loss of resting tone) and during motion (0–6 points; 0 = normal to 6 = no motion), separately. Clinically, the palsy was defined as complete if the patient presented with a complete loss of motor function in the affected hemiface or if the palsy deteriorated to a complete palsy during the inpatient course of treatment. Otherwise, the palsy was defined as incomplete palsy.

#### Electrodiagnostics

The electrodiagnostic tests to evaluate the facial motor function are described in detail elsewhere [12]. Briefly, baseline electroneurography (ENoG), blink reflex testing (BR) and needle electromyography (EMG) were performed as early as possible. If first EMG was performed earlier than 14 days after onset, the examination was repeated at least one time and later than 14 days after onset. The following categorized diagnostic criteria were evaluated: ENoG, peak-to-peak amplitude loss of > 30% (yes = pathologic/no); BR, no R1 and R2 responses ipsilateral (yes/no); EMG, pathological spontaneous activity (yes/no), only single fiber activity or no activity during voluntary EMG (yes/no).

#### Additional functional tests

The other facial nerve function tests were categorized as follows: Ipsilateral stapedius reflex test normal (yes/no); complete loss of gustatory function on ipsilateral side using three-drop-test (yes/no); lacrimal function by Schirmer test (< 10 mm/ ≥ 10 mm); vestibular function represented by normal caloric stimulation (yes/no), and pure tone audiometry for hearing test (normal/ipsilateral hearing loss).

Follow-up examinations included always an otorhinolaryngologic and an EMG examination. Two physicians examined all patients. The first follow-up examination was performed after 6 weeks and then every 3 months until complete recovery. In case of incomplete recovery, recovery with defective healing, the patients were followed in 3-month intervals until pathological spontaneous activity disappeared and/or the patients did not show any improvement for further 3 months.

### Statistical analysis

**Fig. 1:**
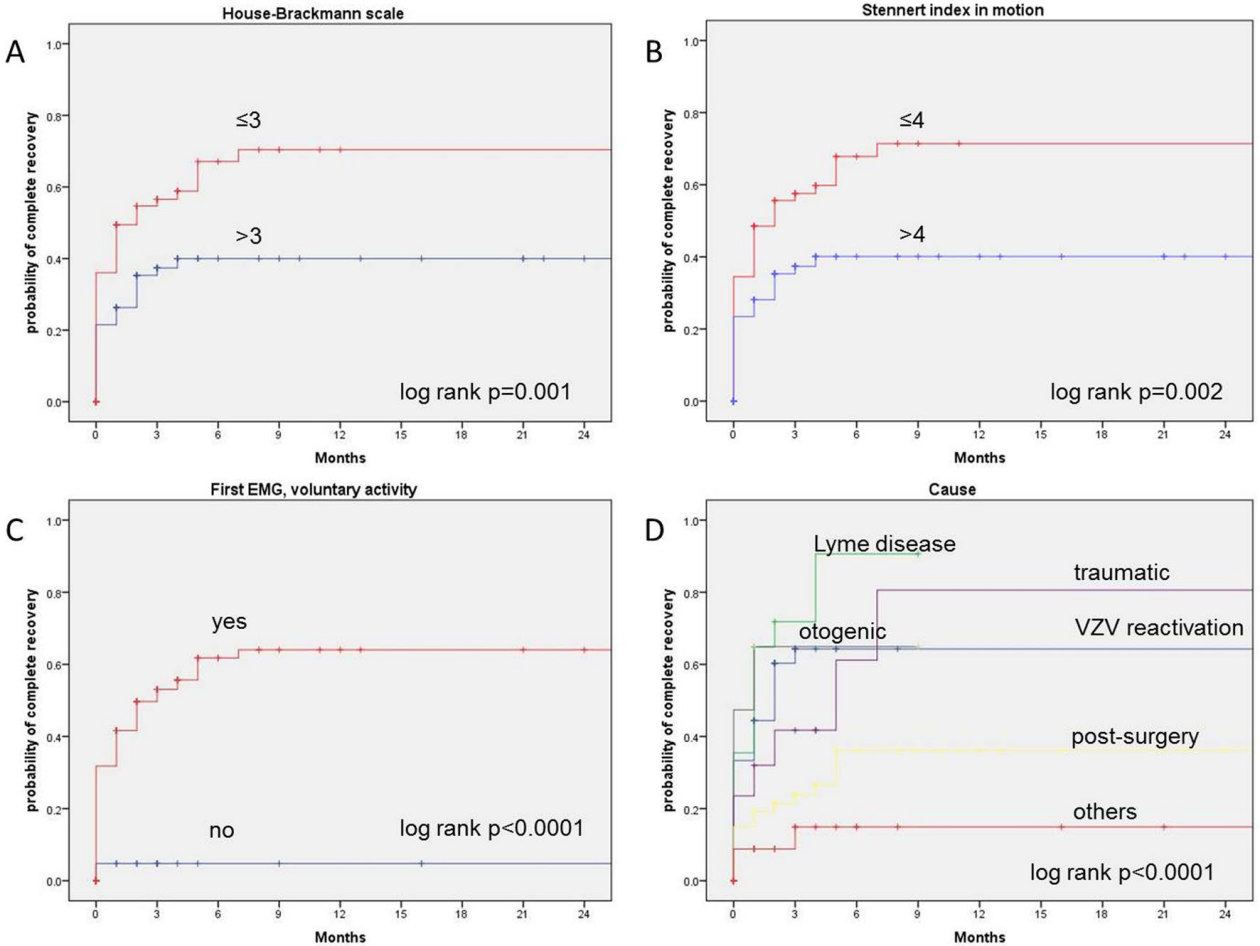
Kaplan–Meier curves showing parameters with prognostic influence on the probability of complete recovery. **a** House-Brackmann scale; **b** stennert index in motion; **c** first EMG, voluntary activity; **d** causes of non-idiopathic FP

If not indicated otherwise, data are presented with mean values ± standard deviation (SD). All statistical analyses were performed using IBM SPSS, version 25. Primary outcome criterion was complete recovery of non-idiopathic FP defined as a grade I on the HB scale indicating normal facial function. Kaplan–Meier curves were constructed to calculate the probability of complete recovery over the time (Fig. 1). The prognostic influence of the diagnostic variables on recovery was analyzed with the log-rank test. Prognostic factors associated with significant impact on complete recovery with a probability value of < 0.05 were included in multivariate analyses using Cox’s proportional hazards model. The hazard ratio of the multivariate Cox regression model indicated the probability of complete recovery. The significance level for the multivariable analyses was set at *p* < 0.05.

## Results

### Patients’ and facial palsy characteristics

264 patients were included. The median age was 57 years. The gender ratio was balanced (47% female and 53% male patients). There was no significant side predominance (48.1% right and 51.9% left side). Two-thirds were classified at baseline to have an incomplete palsy (59.5%) and one-third a complete palsy (31.4%). The median score on HB scale at baseline was III. The median Stennert indexes at rest and during voluntary activity at baseline were 2 and 4, respectively. 17% of the patients had a diabetes mellitus. The most often reason for a non-idiopathic FP was a herpes zoster oticus (36.4%). FP was occurred after surgery in 25.4% (mainly vestibular schwannoma surgery: 9.5%; parotid surgery: 8.0%) of the cases. Lyme disease (11.7%), otogenic reasons (7.2%), trauma (6.4%) and other diagnoses (12.9%) were other reasons for non-idiopathic FP (Table 1). Patients with trauma, often after temporal bone fracture with late-onset-palsy, as underlying disease had the highest rate complete FP (52.9%, *p* = 0.016), i.e. the highest rate of severe cases. In connection with this, traumatic FP was associated more often with pathology spontaneous activity in the first EMG examination (35.3%, *p* < 0.0001) and less often with voluntary activity in the first EMG examination (58.8%) in comparison to the other reasons (Table 1).

**Table 1:** Patients’ characteristics of non-idiopathic facial palsy (*n* = 264)

Parameter	*N*	%	*N*	%	*N*	%	*N*	%	*N*	%	*N*	%	*N*	%	*p* value
Cause of facial palsy	All (*n* = 264)	100	Varicella zoster virus (*n* = 96)	36.4	Lyme disease (*n* = 31)	11.7	Otogenic (*n* = 19)	7.2	Traumatic (*n* = 17)	6.4	Post-surgery (*n* = 67)	25.4	Others (*n* = 34)	12.9	
Gender															Others vs. all *p* = 0.028
Female	124	47.0	50	52.1	11	35.5	9	47.4	7	41.2	37	55.2	10	29.4	
Male	140	53.0	46	47.9	20	64.5	10	52.6	10	58.8	30	44.8	24	70.6	
Affected side															n.s
Right	127	48.1	43	44.8	15	48.4	10	52.6	9	52.9	32	47.8	18	52.9	
Left	137	51.9	53	55.2	16	51.6	9	47.4	8	47.1	35	52.2	16	47.1	
Severity at baseline															Traumatic vs. all *p* = 0.016
Complete palsy	83	31.4	27	28.1	7	22.6	5	26.3	9	52.9	23	34.3	12	35.3	
Incomplete palsy	157	59.5	69	71.9	24	77.4	11	57.9	5	29.4	33	49.3	15	44.1	
Unknown	24	9.1	–	–	–	–	3	15.8	3	17.6	11	16.4	7	20.6	
Diabetes mellitus															VZV vs. all *p* = 0.019
Yes	45	17.0	24	25.0	2	6.5	1	5.3	2	11.8	11	16.4	5	14.7	
No	208	78.8	72	75.0	29	93.5	17	89.5	13	76.5	53	79.1	24	70.6	
Unknown	11	4.2	–	–	–	–	1	5.3	2	11.8	3	4.5	5	14.7	
House-Brackmann scale at baseline															n.s
I	2	0.8	1	1.0	–	–	1	5.3	–	–	–	–	–	–	
II	50	18.9	13	13.5	7	22.6	5	26.3	3	17.6	19	28.4	3	8.8	
III	70	26.5	30	31.3	14	45.2	4	21.1	5	29.4	8	11.9	9	26.5	
IV	78	29.5	45	46.9	6	19.4	4	21.1	3	17.6	14	20.9	6	17.6	
V	28	10.6	6	6.3	4	12.9	2	10.5	2	11.8	6	9.0	8	23.5	
VI	1	0.4	–	–	–	–	–	–	1	5.9	–	–	–	–	
Unknown	35	13.3	1	1.0	–	–	3	15.8	3	17.6	20	29.9	8	23.5	
First EMG; pathological spontaneous activity															VZV vs. all *p* = 0.036; Traumatic vs. all *p* < 0.0001
No			79	82.3	28	90.3	15	78.9	7	41.2	42	62.7	16	47.1	
Yes			6	6.3	2	6.5	1	5.3	6	35.3	9	13.4	4	11.8	
Missing			11	11.5	1	3.2	3	15.8	4	23.5	16	23.9	14	41.2	
First EMG; voluntary activity															n.s
No			6	6.3	-	-	2	10.5	3	17.6	7	10.4	3	8.8	
Yes			79	82.3	30	96.8	14	73.7	10	58.8	42	62.7	17	50.0	
Missing			11	11.5	1	3.2	3	15.8	4	23.5	18	26.9	14	41.2	

Parameter	Mean ± SD	Median, range	Mean ± SD	Median, range	Mean ± SD	Median, range	Mean ± SD	Median, range	Mean ± SD	Median, range	Mean ± SD	Median, range	Mean ± SD	Median, range	*p* value
Age, years	54.5 ± 19.8	57, 1–97	56.3 ± 19.7	60, 20–93	56.1 ± 20.6	63, 10–91	42.1 ± 22.2	40, 1–85	50.1 ± 22.6	49, 11–84	56.4 ± 17.2	58, 23–97	52.8 ± 19.7	55, 4–86	Otogenic vs. all *p* = 0.005
Interval onset to prednisolone therapy, days	2.7 ± 4.2	1.0, 0–24	2.5 ± 4.0	1, 0–24	2.2 ± 2.8	1, 0–13	3.2 ± 4.1	1, 0–14	5.6 ± 6.2	3, 0–20	2.8 ± 5.6	0, 0–20	2.7 ± 3.9	0.5, 0–11	Traumatic vs. all *p* = 0.038
House-Brackmann scale, baseline	3.4 ± 1.0	3, 1–6	3.4 ± 0.8	4, 1–5	3.2 ± 1.0	3, 2–5	3.1 ± 1.2	3, 1–5	3.5 ± 1.2	3, 2–6	3.2 ± 1.1	3, 2–5	3.7 ± 1.0	4, 2–5	Others vs. all *p* = 0.045
Stennert index at rest, baseline	1.9 ± 1.3	2, 0–4	1.9 ± 1.1	2, 0–4	1.7 ± 1.2	1, 0–4	1.5 ± 1.3	1, 0–4	2.2 ± 1.3	2, 0–4	1.9 ± 1.4	2, 0–4	2.0 ± 1.4	2, 0–4	n.s
Stennert index in motion, baseline	4.1 ± 1.8	4, 0–6	4.2 ± 1.7	4, 0–6	4.2 ± 1.6	4, 1–6	3.5 ± 1.6	3, 1–6	4.2 ± 1.7	4.5, 2–6	3.8 ± 2.0	4, 1–6	4.6 ± 1.8	6, 1–6	n.s
Stennert index, total, baseline	6.0 ± 2.8	6, 0–10	6.1 ± 2.6	7, 0–10	5.8 ± 2.5	6, 1–10	5.0 ± 2.8	5, 1–10	6.4 ± 3.0	7, 2–10	5.6 ± 3.3	6, 1–10	6.6 ± 2.9	7, 1–10	n.s

70.5% of the patients received a prednisolone therapy. In traumatic FP, the interval between start of prednisolone therapy and onset of FP was longer (5.6 ± 6.2 days, *p* = 0.038) than in other causes. Patients with otogenic FP were younger (42.1 ± 22.2 years, *p* = 0.005) than the others (Table 1).

On average, House-Brackmann scale and the Stennert index at rest improved after the mean follow-up time of 9.6 ± 18.0 months (*p* < 0.001, Table S1).

### Influence of patients’ and diagnostic results on the recovery time

The median recovery time was 5 months. 39.8% showed a complete recovery, 32.2% a partial recovery and 9.8% no recovery, respectively (18.2% data missed). Higher initial House-Brackmann grading (*p* = 0.001), higher initial Stennert index in motion (*p* = 0.002), no voluntary activity in first EMG (*p* < 0.0001) and the some causes of FP (*p* < 0.0001) were negative predictors for complete recovery (Table S2).

### Influence of patients’ diagnosis on the recovery time

Patients with reactivation of VZV, Lyme neuroborreliosis or otogenic peripheral FP had a significant higher recovery rate (*p* = 0.002, *p* < 0.0001, *p* = 0.018), whereas patients with FP after surgery and other reasons had a significant lower recovery rate (*p* < 0.0001, Table S3).

### Multivariate analysis

After multivariate analyses, voluntary activity in first EMG, Lyme disease and post-surgery cause were identified as independent diagnostic and prognostic factors on the probability of complete recovery (Table 2), in which Lyme disease is associated with better and post-surgery cause with worse recovery rate.

**Table 2:** Multivariate Cox regression analyses on independent diagnostic and prognostic factors on the probability of complete recovery

Parameter	Hazard ratio	95% CI lower	95% CI upper	*p* value*
Model 1				
First EMG; voluntary activity				**0.039**
No	1			
Yes	8.156	1.115	59.651	
House-Brackmann scale (median)				0.916
> 3	1			
≤ 3	1.042	0.489	2.218	
Stennert index, in motion (median)				0.280
> 4	1			
≤ 4	1.499	0.719	3.124	
Varicella zoster reactivation				0.183
No	1			
Yes	1.349	0.868	2.096	
Model 2				
First EMG; voluntary activity				**0.037**
No	1			
Yes	8.284	1.134	60.522	
House-Brackmann scale (median)				0.685
> 3	1			
≤ 3	0.856	0.405	1.811	
Stennert index, in motion (median)				0.153
> 4	1			
≤ 4	1.711	0.820	3.570	
Lyme disease				**0.035**
No	1			
Yes	1.752	1.040	2.951	
Model 3				
First EMG; voluntary activity				**0.044**
No	1			
Yes	7.708	1.057	56.187	
House-Brackmann scale (median)				1.000
> 3	1			
≤ 3	1.000	0.464	2.156	
Stennert index, in motion (median)				0.261
> 4	1			
≤ 4	1.543	0.724	3.288	
Post-surgery palsy				**0.022**
No	1			
Yes	0.460	0.236	0.896	

*CI* confidence interval

*Significant *p* values in bold

## Discussion

### Synopsis of key/new findings

In this presented cohort register-based study on prognostic factors for complete recovery of non-idiopathic FP the most often reason for a non-idiopathic peripheral FP was an infection with Varicella Zoster Virus (36.4%). In 25.4% of the cases, FP was occurred after surgery. Lyme disease (11.7%), otogenic (7.2%), traumatic (6.4%) and other diagnosis (12.9%) were less frequently reasons. Traumatic reasons had a higher proportion of complete facial palsies (52.9%), associated more often with pathology spontaneous activity in the first EMG examination (35.3%) and less often with voluntary activity in the first EMG examination (58.8%). Furthermore, in traumatic FP the interval with onset of prednisolone therapy was longer (5.6 ± 6.2 days). After analysis the recovery time to normal facial function the onset of prednisolone therapy, the House-Brackmann scale at begin, the Stennert index in motion at begin, voluntary activity in first EMG and cause of FP had influence on the recovery time. Patients with infection with VZV, Lyme neuroborreliosis or otogenic peripheral FP had a significant higher recovery rate in comparison to non-idiopathic FP, whereas patients with FP after surgery and other reasons had a significant lower recovery rate. Lyme disease is associated with better and post-surgery cause with worse recovery rate.

### Strengths and weaknesses of the study

A strength of the study is the consequent registration of all new patients with non-idiopathic peripheral FP treated in one tertiary university hospital in a register with standardized data set since 2007. All cases were treated together by the department of otorhinolaryngology and the department of neurology in the Facial Nerve Center of the university hospital. Another strength is the comprehensive diagnostic work-up (not only for prognostic reasons but primarily also for differential diagnostics) including the most relevant electrodiagnostic tests and other tests originally used as topodiagnostic tests in the era before introduction of high-resolution radiologic imaging. As facial grading is subjective and has a limited reliability [13], it is important to notice that EMG was performed as a standard diagnostic tool, too. EMG allows for an objective measurement of facial muscle function [12].The importance was confirmed by the present study. EMG was more reliable to predict outcome than facial grading.

Patients were not treated within a randomized prospective clinical trial. Therefore, a selection bias cannot be ruled out. The duration of follow-up of the patients was variable. No defined times of investigation during the follow-up for all patients, like in a prospective clinical trial, were available. To overcome this limitation, Kaplan–Meier statistics were used to determine the probability of complete recovery over time.

### Comparisons with other studies

It is important to compare the outcome of non-idiopathic FP to the outcome of idiopathic FP: EMG evaluation for pathological spontaneous activity is in non-idiopathic FP and also in Bell`s palsy [14] an important prognostic factor for recovery time from acute facial palsy. Volk et al. identified moreover start of treatment, grading and stapedius reflex test. In this study, otogenic cause (87%), Lyme disease (78%), VZV reactivation (77%) had a slightly better probability of recovery at 9 months after onset in comparison to Bell`s palsy (73%). Traumatic cause had a worse outcome (46%) [14]. Furthermore, Urban et al. identified severity of the palsy, facial electrodiagnostics and stapedius reflex testing are the most powerful tool for prognostication of recovery time after Bell’s palsy. Prednisolone therapy, which started within a time window of 96 h after onset, had the highest probability of complete recovery [15].

Chang et al. analyzed the characteristics of patients with acute peripheral FP showing Varicella Zoster Virus DNA in saliva [16] and showed, that VZV DNA-positive patients had worse hearing and incomplete recovery compared to VZV DNA-negative patients. In our collective, patients with VZV infection had significant higher recovery rate in comparison to all other non-idiopathic facial palsies. Morishima et al. evaluated 2013 prognostic factors of synkinesis following Bell’s palsy and Ramsay Hunt syndrome [17]. The lowest Yanagihara score, the change in Yanagihara score after 1 month and ENoG value at the onset were found to be important factors for predicting synkinesis. Takemoto et al. identified 2011 ENoG as the most effective factor for prediction of the prognosis of peripheral FP in case of Bell`s palsy or Ramsay Hunt syndrome, and more than 85% degeneration had the best specificity and sensitivity to predict non-recovery [18]. In our study, voluntary activity in first EMG was the most valid independent factor for recovery. Kim et al. and Cai et al. realized that patients with Ramsay Hunt syndrome had a significant unfavorable recovery rate and poorer prognosis than patients with Bell’s palsy [19, 20]. In our collective, patients with Ramsay Hunt syndrome had the same recovery rate as patients with Bell´s palsy.

Jowett et al. demonstrated, that there was an association between corticosteroid use in acute Lyme disease associated FP and worse long-term facial function outcomes [21]. In our opinion, nevertheless the cause of non-idiopathic FP a fast treatment with prednisolone after onset could be useful and essential.

Ping et al. analyzed possible prognosis factors of systemic steroid in managing traumatic facial nerve palsy after a blunt craniofacial injury retrospectively [22]. The outcome showed steroid therapy onset within 24 h and steroid therapeutic duration for longer than 14 days possessed a significantly better recovery rate. In our study, steroid started late and could potentially had influence on the recovery rate. Yadav et al. evaluated the outcome of conservative management in traumatic facial paralysis with regard to type of trauma, onset and electrodiagnostic tests [23]. Patients with incomplete FP are candidates for conservative management and also patients with complete facial paralysis for up to 3 months even in cases where ENoG and NET suggest poor prognosis. In our study, patients with traumatic peripheral FP had no significant worse recovery rate than patients with other causes of non-idiopathic FP. Thakar et al. concluded 2018, that for undisplaced temporal bone fractures, nonsurgical treatment leads to near complete recovery and is superior to reported surgical results [24]. Surgical exploration should not be first-line treatment for undisplaced longitudinal temporal bone fractures associated with complete facial nerve paralysis and unfavorable electrophysiological features. In our opinion, surgery remains reserved for special cases. In most cases, conservative therapy with fast onset of therapy with prednisolone is sufficient. An immediate total facial paralysis associated with a clear-cut fracture going through the Fallopian canal (Canalis nervi facialis) is perhaps the only case requiring surgery after traumatic FP in cause of temporale bone fracture [25]. In case of direct cut through branches of facial nerve, a fast reconstruction is necessary and the therapy of first choice [7, 14].

Especially early-onset FP after vestibular schwannoma surgery had a bad prognosis of recovery [26]. In case of post-surgery FP, spontaneous activity and voluntary activity in first EMG could be an essential tool to differentiate patients’ prognosis. In our opinion and experience, if there is no recovery within 1 year after surgery a facial nerve reconstruction should be considered.

### Clinical applicability of the study

Traumatic reasons of non-idiopathic peripheral FP seem to be associated with severe disease histories. The higher proportion of diagnosis of degenerative axonal lesion could be one point of worse outcome. Furthermore, the late onset of prednisolone therapy could be another. Especially in case of traumatic reasons, a fast onset of prednisolone therapy and/or facial nerve decompression by surgery should be considered. It would be worthwhile to analyze if patients with worse prognosis would profit from an intensified therapy (higher prednisolone dose). The addition of the EMG result is very helpful to identify as early as possible the patients with high risk for incomplete recovery and development of synkinesis. Because of the functional and psychosocial impairments that might occur, these patients need early education about treatment options, and short follow-up visit intervals. Still many patients with facial synkinesis are undertreated. High-risk patients might profit from an early referral to a specialist center for facial nerve care [27].

## Conclusion

In our study infectious causes for non-idiopathic FP like VZV reactivation and Lyme disease had best probability for complete recovery. Post-surgery FP had a worse prognosis.

## Electronic supplementary material

Below is the link to the electronic supplementary material.

Supplementary file1 (DOCX 21 kb)

## Data Availability

All authors had full access to all of the data in the study. OGL takes responsibility for the integrity of the data and the accuracy of the data analysis. No additional data available.
