# Supplementary material for: Non-idiopathic peripheral facial palsy: prognostic factors for outcome
Source: Eur Arch Otorhinolaryngol. 2020 Oct 6;278(9):3227–35. doi: 10.1007/s00405-020-06398-6 (PMC8328849; doi:10.1007/s00405-020-06398-6)
Supplement: Supplementary file 1 — Supplementary file1 (DOCX 21 kb) [file 405_2020_6398_MOESM1_ESM.docx]

**Supplement Tables**

**Table S1**

| **Table S1** Facial nerve grading at admission and at last examination during follow-up (n = 229; n = 35 lost during follow-up)* | | | |
| --- | --- | --- | --- |
| **Parameter** | **At admission** | **Last examination** |  |
|  | **Mean±SD** | **Mean±SD** | ***p* value**** |
| House-Brackmann scale | 3.36±1.00 | 2.15±1.18 | **<0.001** |
| Stennert index at rest | 1.87±1.25 | 0.75±1.13 | **<0.001** |
| Stennert index in motion | 4.12±1.76 | 1.79±2.01 | **0.015** |
| Stennert index, total | 5.97±2.78 | 2.54±3.01 | **0.003** |

*with a mean follow-up time of 9.6±18.0 months, **significant *p* values in bold.

**Table S2**

| **Table S2** Influence of patients’ and diagnostic results on the recovery time | | | |
| --- | --- | --- | --- |
| **Parameter** | **Median recovery time (months)** | **95% CI**  **(months)** | **Log rank test**  ***p* value*** |
| All patients | 5.05 | 0.0-27.8 |  |
| Gender  Female  Male | 43.81  4.08 | 0.0-100.3  1.3-6.7 | 0.828 |
| Side  Right  Left | 5.27  5.04 | 0.0-29.7  1.9-8.1 | 0.382 |
| Age (Median)  < 57 years  ≥ 57 years | 5.37  5.22 | 0.0.-19.8  0.0-28.7 | 0.547 |
| Diabetes mellitus  No  Yes | 5.78  NA | 2.6-7.4  NA | 0.598 |
| Onset prednisolone therapy  ≤24 hours  25-48 hours  49-72 hours  73-96 hours  97-120 hours  Over 120 hours | 2.17  1.48  3.89  0.44  NA  4.32 | 1.1-2.9  NA  0.0-6.6  NA  NA  0.0-9.3 | 0.196 |
| Severity at baseline  Incomplete palsy  Complete palsy | 4.38  NA | 1.7-6.3  NA | 0.375 |
| House-Brackmann scale (Median)  ≤3  >3 | 2.21  NA | 0.4-3.6  NA | **0.001** |
| Stennert index, at rest (Median)  ≤2  >2 | 3.42  NA | 1.0-5.0  NA | 0.211 |
| Stennert index, in motion (Median)  ≤4  >4 | 2.40  NA | 0.9-3.1  NA | **0.002** |
| Stennert index, total (Median)  ≤6  >6 | 2.56  NA | 0.0-4.4  NA | 0.077 |
| First EMG; pathological spontaneous activity  No  Yes | 4.36  NA | 2.2-5.8  NA | 0.283 |
| First EMG; voluntary activity  No  Yes | NA  3.33 | NA  1.1-4.9 | **<0.0001** |
| ENG ipsilateral normal  No  Yes | 1.08  1.46 | 0.0-2.6  0.0-2.1 | 0.766 |
| Blink reflex R1/R2 ipsilateral  No  Yes | 2.39  2.17 | 0.7-3.3  0.0-5.0 | 0.366 |
| Schirmer test normal  No  Yes | 4.39  1.07 | 0.3-7.7  0.1-1.9 | 0.530 |
| Stapedius reflex ipsilateral  No  Yes | 3.18  1.59 | 0.7-5.3  0.0-2.1 | 0.398 |
| Taste function normal  No  Yes | 2.73  2.71 | 0.3-3.7  0.0-4.6 | 0.809 |
| Hearing test normal  No  Yes | 4.74  5.71 | 1.9-6.1  0.0-43.7 | 0.467 |
| Vestibular function normal  No  Yes | 7.10  2.93 | NA  1.2-2.8 | 0.084 |
| Cause  Varicella zoster  Lyme disease  Otogenic  Traumatic  Post-surgery  Other | 2.63  1.90  1.68  5.82  NA  NA | 1.1-2.9  0.3-1.7  NA  0.0-10.5  NA  NA | **<0.0001** |

CI = confidence interval; *significant *p* values in bold. NA = not applicable, because the overall probability to recovery was less than 0.5 in this subgroup.

**Table S3**

| **Table S3** Influence of patients’ diagnosis on the recovery time | | | |
| --- | --- | --- | --- |
| **Parameter** | **Median recovery time (months)** | **95% CI**  **(months)** | **Log rank test**  ***p* value*** |
| All patients | 7.0 | 0.0-18.6 |  |
| Varicella Zoster Virus  Yes  No | 3.0  24.0 | 2.1-3.9  7.2-40.8 | **0.002** |
| Lyme neuroborreliosis  Yes  No | 2.0  12.0 | 1.1-2.9  0.0-40.1 | **<0.0001** |
| Otogenic  Yes  No | 1.0  7.0 | 0.0-2.3  0.0-18.8 | **0.018** |
| Traumatic  Yes  No | 4.0  8.0 | 1.0-7.0  0.0-19.7 | 0.702 |
| Post-surgery  Yes  No | 99.0  3.0 | NA  1.7-4.3 | **<0.0001** |
| Others  Yes  No | NA  5.0 | NA  0.0-18.6 | **<0.0001** |

CI = confidence interval; *significant *p* values in bold. NA = not applicable, because the overall probability to recovery was less than 0.5 in this subgroup.
